# Multi‐Omics Analyses Reveal the Red and Far‐Red Light Combination Enhancing Heterologous Protein and Metabolite Production in *Nicotiana benthamiana*

**DOI:** 10.1111/pce.15573

**Published:** 2025-04-23

**Authors:** Yating Zhang, Fengjiao Wang, Ran Du, Tao Li, Shaoqun Zhou, Jianbin Yan, Wei Li

**Affiliations:** ^1^ Shenzhen Branch, Guangdong Laboratory of Lingnan Modern Agriculture, Key Laboratory of Synthetic Biology Ministry of Agriculture and Rural Affairs, Agricultural Genomics Institute at Shenzhen, Chinese Academy of Agricultural Sciences Shenzhen China; ^2^ College of Plant Science and Technology Huazhong Agricultural University Wuhan China; ^3^ Institute of Environment and Sustainable Development in Agriculture Chinese Academy of Agriculture Sciences Beijing China

**Keywords:** agroinfiltration, expression efficiency, light quality, *Nicotiana benthamiana*, synthetic biology

## Abstract

Transient expression of exogenous protein in *Nicotiana benthamiana* leaves via agroinfiltration offers a rapid and efficient platform for functional gene discovery and heterologous production of valuable eukaryotic proteins and metabolites. Though light quality is an important factor for plant photomorphogenesis, its impact on the efficiency of transient expression remains unexplored. In this study, we examined the influence of five representative light qualities with varying wavelength mix on the *N. benthamiana* growth and recombinant green fluorescent protein (GFP) production. Plants with red and far‐red light treatment (LED‐red) showed the highest GFP expression, 57.4% higher than white light. Further study showed that a higher dosage of post‐infiltration *Agrobacterium* and the resulting increase in the number of transcripts contribute to the expression rate enhancement. Moreover, as for exogenous metabolites, a 76.5% increase of accumulated taxadiene was also observed in LED‐red group. Integrated transcriptomic, proteomic and metabolomic revealed that LED‐red plants reduced the resistance pathways before infiltration, inducing a higher dosage of post‐agroinfiltration *Agrobacterium*. Our results suggest that *N. benthamiana* grown under LED‐red creates a more favorable environment for *Agrobacterium* growth, enhancing heterologous protein and metabolite production. This study highlights the potential utilization of light quality as an implementable tool in plant synthetic biology.

## Summary statement

Red and far‐red light induced the highest heterologous expression efficiency, which depended on a higher dosage of post‐agroinfiltration *Agrobacterium* and resulted in increased transgene transcripts.

## 1 Introduction

Plants represent a sustainable and powerful chassis for synthetic biology, as their growth relies on low‐cost resources: atmospheric carbon, sunlight, soil and unsterilized water, compared to microbial fermentation (Liu et al. 2022). *Nicotiana benthamiana* is a favorable bioreactor for transient transformation because of its substantial biomass, large leaves and a short growth cycle (Holtz et al. 2015), and more importantly, its high susceptibility to *Agrobacterium tumefaciens* and plant viruses (Goodin et al. 2008).

*Agrobacterium*‐mediated transient transformation in *N. benthamiana* is a highly efficient, simple and consistent technique, accelerating plant functional genetics research and heterologous production of valuable eukaryotic proteins (Kado 2014; Guo et al. 2019). *Agrobacterium* carrying plasmids enters the plant through pressure infiltration. As one of the most widely used plasmids in metabolic engineering research, pEAQ‐HT harbors an RNA silencing suppressor *P19* gene from tombusviruses, facilitating the efficient and rapid transformation of target genes while utilizing plants as a eukaryotic alternative for heterologous production (Sainsbury et al. 2009). In this process, the ‘disarmed’ transfer DNA (T‐DNA) on plasmid deprived from the tumor‐inducing genes that cause crown gall disease are engineered with the genes of interest. Transgene expression usually peaks between 3 and 5 days post‐infiltration and the leaves can be harvested for functional genetic studies or product extraction (Pitzschke 2013; Hwang et al. 2017). This platform has been employed for the elucidation and reconstruction of metabolic pathways of numerous valuable plant‐derived natural compounds, such as kavalactone (Pluskal et al. 2019), colchicine (Nett et al. 2020), strychnine (Hong et al. 2022), limonoids (De‐La‐Peña et al. 2023), as well as paclitaxel (Jiang et al. 2024), and so on.

Extensive research efforts have optimized the efficiency of different steps involved in the exogenous expression process in the *Agrobacterium*‐mediated transient transformation in plant chassis. Among multiple participants including chassis plant, *Agrobacterium* and plasmid in the system, the effects of environmental factors on the transient system are manifold. For example, temperature has been wildly reported to control T‐DNA transfer efficiency and high temperature resulted in relatively higher accumulations of recombinant proteins (Fullner and Nester 1996; Dillen et al. 1997; Jung et al. 2015; Matsuda et al. 2019). Light as one of the most important environmental factors dominates plant photosynthesis and photomorphogenesis. Growth increment of chassis plants controlled by high light intensity was reported to enhance the recombinant protein yield (Matsuda et al. 2019). However, another research revealed that the low light intensity before the agroinfiltration could improve the heterologous protein accumulation in *N. benthamiana* (Zhang et al. 2023). These results showed that the light environments would affect the *Agrobacterium*‐mediated transient transformation efficiency, by changing the state of the chassis plant.

Plants can adjust their growth in response to light quality by various photoreceptors, ranging from ultraviolet light (280–400 nm), blue light (400–500 nm), red light (600–700 nm) to far‐red light (700–750 nm) (Lin 2000; Demotes‐Mainard et al. 2016; Neugart and Schreiner 2018; Christie and Zurbriggen 2021). Plants showed dysfunctional photosynthesis under monochromatic red light, which can be reversed by blue light (Hogewoning et al. 2010; Trouwborst et al. 2016). In addition to contributing to photosynthesis, blue light typically promotes flavonoids and phenolics accumulation (Taulavuori et al. 2016, 2018). UVB light and far‐red light are inefficient spectra for photosynthesis, while UVB light signal induces dwarf plant size (Qian et al. 2021) and far‐red light participates in regulating stem and leaf blade elongation (Demotes‐Mainard et al. 2016). Researchers utilize monochromatic light signals for optogenetics (Emiliani et al. 2022) or combine different light qualities to manipulate crop growth and defense (Jones 2018; Lazzarin et al. 2021). Despite its potential importance, the impact of light quality on heterogeneous biosynthesis in plant chassis remains largely unexplored. Furthermore, whether the modulating of the light quality environment can enhance the efficiency of this process remains to be investigated.

For the enhancement of transgene efficiency, researchers have found that reducing the plant immune responses to *A. tumefaciens* infection promote the heterologous biosynthesis (Zipfel et al. 2006; Rosas‐Díaz et al. 2017). Researches show that *Arabidopsis thaliana* mutants deficient in either *ics1* (exhibiting reduced salicylic acid [SA] level) or *NPR1* (lacking SA signal transduction) displayed heightened efficiency in *Agrobacterium*‐mediated transient expression (Rosas‐Díaz et al. 2017; Zhai et al. 2017). Light quality is involved in the regulation of plant immune function. For instance, impaired photosynthesis has been linked to disrupted response to pathogen immunity (Manfre et al. 2011; Göhre et al. 2012). Furthermore, light photoreceptors are reported to regulate *Pseudomonas syringae* resistance through interactions with the SA signaling pathway (Genoud et al. 2002; Griebel and Zeier 2008).

Herein, we investigated the influence of five representative light qualities on the regulation of growth states of *N. benthamiana* chassis and the expression efficiency of heterologous protein. Our result demonstrated that red and far‐red light treatment induced the highest expression efficiency of tested GFP protein compared with other light qualities. This effect depended on a higher dosage of post‐agroinfiltration *Agrobacterium*, which resulted in the increased number of transgenes transcripts. We further verified that light quality optimization is conducive to the synthesis of heterologous products such as taxadiene. Integrated transcriptomic, proteomic and metabolomic confirmed that plant immunity may be the key factor for efficiency elevation regulated by light quality. Overall, we discovered a practical light‐quality system for higher protein and metabolite production in *N. benthamiana* chassis.

## 2 Material and Methods

### 2.1 Plant Materials and Light Treatments

*N. benthamiana* were germinated in rockwool plugs (Grodan, Roermond, the Netherlands) under fluorescent white light with 100 μmol m^−2^ s^−1^ photon flux density (PFD). Fourteen days after sowing, the second true leaf unfolding seedlings were transferred to rockwool cubes (7.5 cm × 7.5 cm × 7 cm; Grodan). In the growth chamber, CO_2_ partial pressure was close to ambient levels, relative humidity maintained at 65%–70%, with day/night temperatures set at 24/21°C, and photoperiod was 16 h (6:00–22:00). Seedlings were regularly irrigated with modified Hoagland nutrient solution (pH = 5.5; EC = 2.0 dS m^−1^).

**Figure 1:**
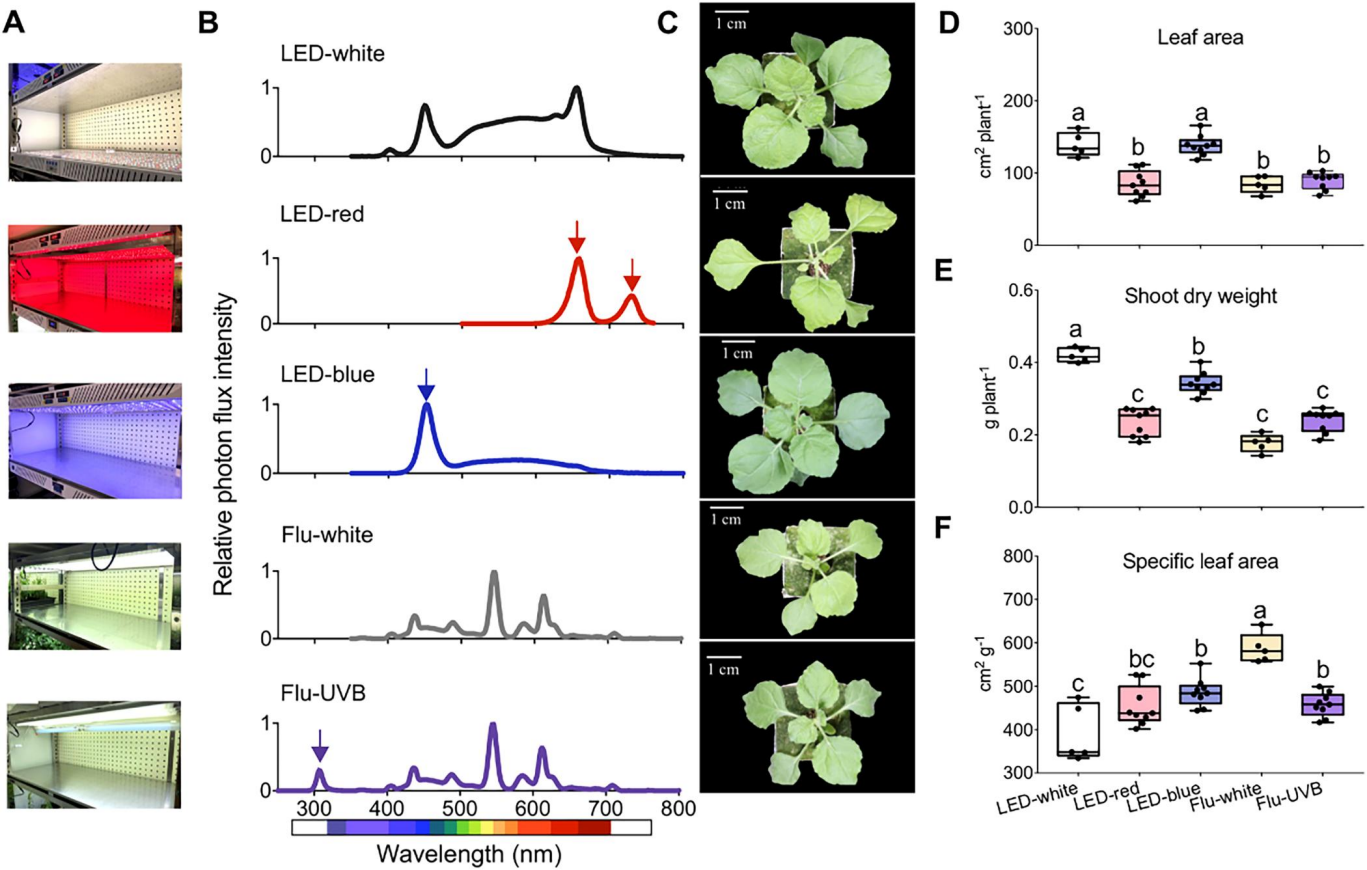
The effect of light qualities on *Nicotiana benthamiana* growth. (A) Growth chamber with different light environments. (B) The spectrum of five light qualities in this study. (C) Plant morphology under different light treatments for 7 days' growth from the top view. Leaf area (D), shoot dry weight (E) and specific leaf area (F) of the plants under different light treatments at 0 dpi; different letters show statistically significant differences (*p* < 0.05).

Dimmable LED tubes (Jiuyu company, Bejing, China) and fluorescent light tubes (Jiuyu company, Bejing, China) were 50 cm above the growth plate, respectively (Figure 1A). PFD at 25 cm above the growth plate was adjusted to ~180 μmol m^−2^ s^−1^ at the center of the cultivation unit, and PFD at five positions per unit was measured (Supporting Information S1: Table S1). Five light treatments were set in this study, which were: (i) fluorescent white light (Flu‐white); (ii) fluorescent white light with UVB light (Flu‐UVB), where UVB light intensity was ~1.5 μmol m^−2^ s^−1^; (iii) a mixture of red and far‐red light (LED‐red); (iv) LED white light (LED‐white); (v) LED blue light (LED‐blue). Peak intensities of UVB/blue/red/far‐red light were 306/450/660/730 nm, respectively (Figure 1B). PFD and light spectra were routinely monitored using a spectroradiometer (Avaspec‐2048CL, Avates, Apeldoorn, The Netherlands).

After the 14‐day seedlings stage, plants were transferred to five light treatments, these plants were grown for another 7 days under different light treatments to be ready for the modified *A. tumefaciens* infiltration. The plants were moved to dark environment for 1 day after the infiltration. The duration for post‐infiltration was 4 days for GFP protein expression and 6 days for taxadiene compound accumulation.

### 2.2 Transient Transgene Assay

#### 2.2.1 Vector Construction and Vacuum Infiltration for Transgene Expression

*Agrobacterium* strains GV3101, carrying GFP and TS1 transgenes cloned into pEAQ‐HT vector, detailed structure and genes (including *P19* and *NeoR_KanR*) information of the plasmid was provided in Supporting Information S1: Figure S11 and Table S3 (Sainsbury et al. 2009), were resuspended in an infiltration solution (10 mM MES (2‐[N‐morphollino]‐ethanesulfonic acid) pH 5.6, 10 mM MgCl_2_, and 100 μM acetosyringone) at OD_600_ = 0.6 for injection into leaves of 3‐week‐old plants. Each treatment in the experiment involved at least six plants, with three leaves per plant.

#### 2.2.2 GFP Fluorescence Intensity Measurement

Leaf samples from infiltrated *N. benthaminana* plants by *Agrobacterium* strain containing the pEAQ‐HT‐GFP plasmid were collected at 4 dpi (days post‐infiltration). Leaf samples were ground into powder in liquid nitrogen using a mortar and pestle. RIPA lysis buffer (P0045, Beyotime Biotechnology, China) with 1 mM PMSF (ST506, Beyotime Biotechnology, China) was used for protein extraction. *N. benthamiana* fluorescence was quantitatively measured using a Spark Multimode Microplate Reader (Tecan, Swiss). The protein extraction using RIPA lysis was placed into the 96‐well black plates (Thomas Scientific 655096). GFP measurement was performed using the following settings: GFP excitation peaks at 385 nm, GFP emission peaks at 512 nm and bandwidth at 5 nm.

#### 2.2.3 qRT‐PCR Assay

For qRT‐PCR analysis of genes on plasmid (primers were provided in Supporting Information S1: Table S2), RNA was isolated using the total RNA isolation kit from Vazyme with a DNA digestion step using Prime Script TMRT reagent Kit with gDNA Eraser (TaKaRa). cDNAs were prepared using TB Green Premix Ex TaqTM II (Tli RNaseH Plus, TaKaRa). Assays were performed using three independent biological replicates. The relative amounts of transcripts for different genes were normalized to tubA transcript levels. The statistical assay (unpaired Student's *t*‐test, two‐tailed option) was performed via GraphPad Prism 10 software (https://www.graphpad.com/scientific‐software/prism/).

#### 2.2.4 Western Blot Analysis

Proteins extracted using RIPA lysis buffer were separated by SDS‐PAGE on a 12% polyacrylamide gel (GenScript, China) and then transferred onto a polyvinylidene difluoride (PVDF) membrane (Immobilon‐P; Millipore) by electroblotting. The proteins were stained with Coomassie blue and analyzed with antibodies: GFP antibody (A11122, Thermo Fisher) was used at a 1:2000 dilution, followed by a secondary peroxidase‐conjugated antibody (AH433, Beyotime Biotechnology, China). Detection was performed using enhanced chemiluminescence (SuperSignal West Femto Maximum Sensitivity Substrate, Thermo Fisher Scientific) on a Gel Imaging Analysis System (GelView 6000 plus, BTL, Guangzhou, China).

#### 2.2.5 Confocal Imaging

Images of GFP overexpression were obtained using a Leica TCS SP5 II microscope, equipped with a 488‐nm argon laser for GFP excitation. Image processing was conducted using the Leica LAS AF Lite platform or the Java‐based ImageJ software (National Institutes of Health). GFP images represent a maximum projection of stacks spaced 7 μm apart along the *z*‐axis.

#### 2.2.6 Taxadiene Quantification

Leaf samples of the pEAQ‐HT‐TS1 plasmid contained *Agrobacterium* strain infiltrated plants were collected at 6 dpi. In each treatment, a random selection of eight plants were selected and divided into four subgroups. Infiltrated fresh leaves from two plants were removed and soaked in 20 mL MTBE containing 100 μM nonadecane as an internal standard in brown glass bottles for 12 h. The organic solvent phases were dried to 1 mL by nitrogen flow. GC‐MS analysis: Samples were redissolved in 1 mL MTBE, and 1 µL of each was directly injected into a Trace1310 GC Ultra gas chromatograph coupled to TSQ9000 mass spectrometer (Thermo Scientific). Separation was performed on a DB‐5Ul column (30 m × 0.25 μm × 0.25 μm thickness) in splitless mode. Ultra‐helium was used as the carrier gas at a flow rate of 1.2 mL/min. The oven temperature was kept constantly at 100°C for 1 min, then increased to 175°C at the increment of 15°C/min, and then increased to 220°C at the increment of 4°C/min, and held for 2 min, and then increased to 290°C at the increment of 20°C/min, and finally held for 5 min. The injector and transfer line temperatures were set at 270°C and 250°C, respectively. The column effluent was ionized by electron impact ionization at 8 and 12 eV. Taxadiene was quantified by multiple reaction monitoring (MRM). The scanned ion pairs of taxadiene included 67.1–95.1, 91.1–107.1 and 107.1–122.2 (quantitative ion pair). 41.0–57.1 and 43.0–71.1 are ion pairs for nonadecane. Taxadienes were quantified by comparing their total ion count peak area with that of the internal standard, nonadecane. For each independent experiment, a minimum of eight plants of per treatment were infiltrated, and the experiment was repeated in three independent runs.

### 2.3 Plant Growth and Physiological Parameters Determination

#### 2.3.1 Leaf Area and Shoot Dry Weight

The *Agrobacterium* strain carrying pEAQ‐HT‐GFP infiltrated and non‐infiltrated *N. benthaminana* plants were destructively measured at 0 dpi and 4 dpi. Shoot dry weight and leaf area were determined. To assess the whole‐plant leaf area, leaves were arranged on a black cloth, and overhead images were captured from a fixed distance. Adobe Photoshop CC (v. 19.1.4) was using to quantify the whole‐plant leaf area, by comparing pixel numbers of the projected leaf area of the whole plant with the pixel numbers of a white card (2 × 2 cm), following the method by Jarou (2009). Plant shoot tissue was dried at 80°C in a ventilated oven (DHG‐9070A, Jinghong, Shanghai, China). Specific leaf area (SLA) was calculated by dividing leaf area by leaf dry weight.

#### 2.3.2 Total Protein Content

The *Agrobacterium* strain carrying pEAQ‐HT‐GFP infiltrated and non‐infiltrated *N. benthaminana* plant leaves were sampled at 0 and 4 dpi. Samples were ground into powder in liquid nitrogen using a mortar and pestle. The RIPA lysis buffer (P0045, Beyotime Biotechnology, China) with 1 mM PMSF (ST506, Beyotime Biotechnology, China) was used for protein extraction from leaf tissue. The protein concentration was determined using BCA protein assay kit (Beyotime Biotechnology, China).

#### 2.3.3 Leaf Pigments Content

Same leaf samples as those used for determination of protein content were used for leaf pigments determined. The leaf powder was stored in 10 mL 95% ethanol, kept at 4°C for 48 h in the dark. The absorbance of the extract was measured at 665, 649 and 470 nm using a UV‐Vis spectrophotometer (UV‐1800, Shimadzu, Japan). Chlorophyll concentrations were calculated using the equations derived by Lichtenthaler and Buschmann (2001).

#### 2.3.4 Bacterial Growth Assay

Samples were taken from the leaves infiltrated by *Agrobacterium* strain carrying pEAQ‐HT‐GFP at 4 dpi using a 10 mm diameter borer. Three leaf disks were collected from each plant and placed in 1 mL of 10 mM MgCl₂. Serial dilutions (100 μL) of the bacterial suspensions obtained were plated on LB agar plates containing rifampicin (50 μg/mL) and kanamycin (50 μg/mL). For each experiment, at least four plants per light treatment and three leaves per plant were used.

### 2.4 Multi‐Omics Analysis

#### 2.4.1 RNA Sequencing and Transcriptome Analysis

*mRNA extraction and library contraction*: The same leaf samples as those used for total protein content determination were used for transcriptome analysis. TRIzol Reagent (Magen) was used for leaf tissue total RNA extraction. RNA quality were assessed using the A260/A280 absorbance ratio (Nanodrop ND‐2000 system, Thermo Scientific, USA) and RIN values of RNA (Agilent Bioanalyzer 4150 system, Agilent Technologies, CA, USA). The mRNA was purified from 1 µg total RNA using oligo (dT) magnetic beads, fragmentated and reverse‐transcribed into cDNA. The cDNA fragments were then adapter‐ligated for the paired‐end library. Adaptor‐ligated cDNA was used for PCR amplification. PCR products were purified (AMPure XP system) and library quality was assessed (Agilent Bioanalyzer 4150 system), and sequenced (Illumina Novaseq. 6000/MGISEQ‐T7).

*Transcriptome of N. benthamiana analysis*: Raw reads of fastq format were firstly processed through fastp (https://github.com/OpenGene/fastp) with the default parameters. Clean reads data were used to map to the *N. benthamiana* genome (PRJCA010759) with STAR (v.2.7.9a, http://www.ccb.jhu.edu/people/infphilo). StringTie (https://ccb.jhu.edu/software/stringtie) was used to count the reads numbers mapped to each gene. Then, TPM (Transcripts Per Kilobase Million) of each gene was calculated based on the length of the gene and the reads count mapped to this gene. Differential expression analysis was performed using the DESeq. 2 (http://bioconductor.org/packages/release/bioc/html/DESeq.2.html), DEGs with |log2FC | > 1 and *p*‐adj < 0.05 were considered to be significantly differential expressed genes (DEGs). ClusterProfiler R software package was used for KEGG pathway enrichment analysis.

*Transcriptome of pEAQ‐HT‐GFP plasmid analysis*: Paired reads that unmapped to the *N. benthamiana* genome in STAR exported bam files were extracted to be mapped to the pEAQ‐HT‐GFP plasmid sequence by STAR. StringTie was used to count the reads numbers mapped to each gene. Then TPM of GFP, p19 and NeoR_KanR was calculated based on the length of the gene and reads count.

#### 2.4.2 4D‐Label Free Peptide Sequencing and Proteome Analysis

The same frozen leaf tissue powders as those used for transcriptome were used for proteome analysis. *Protein extraction and digestion*. SDT (4% SDS, 100 mM Tris‐HCl, pH 7.6) buffer was used for leaf sample lysis and protein extraction. The amount of protein was quantified with the BCA Protein Assay Kit (Bio‐Rad, USA). Twenty micrograms of protein for each sample were mixed with 5X loading buffer respectively and boiled for 5 min. The proteins were separated on 4%–20% SDS‐PAGE gel. Protein digestion by trypsin was performed according to the filter‐aided sample preparation (FASP) procedure described by Matthias Mann. The digest peptides of each sample were desalted on C18 Cartridges (Empore SPE Cartridges C18 (standard density), bed I.D. 7 mm, volume 3 mL, Sigma), concentrated by vacuum centrifugation and reconstituted in 40 µL of 0.1% (v/v) formic acid.

*FASP digestion procedure*. DTT was added to each sample respectively and mixed at 600 rpm for 1.5 h (37°C). After the samples cooled to room temperature, IAA was added with the final concentration of 20 mM into the mixture to block reduced cysteine residues and the samples were incubated for 30 min in darkness. Next, the samples were transferred to the filters respectively. The filters were washed with 100 μL UA buffer three times and then 100 μL 25 mM NH_4_HCO_3_ buffer twice. Finally, trypsin was added to the samples (the trypsin: protein [wt/wt] ratio was 1:50) and incubated at 37°C for 18 h, and the resulting peptides were collected as a filtrate. The peptides of each sample were desalted on C18 Cartridges (Empore SPE Cartridges C18 [standard density], bed I.D. 7 mm, volume 3 mL, Sigma), concentrated by vacuum centrifugation and reconstituted in 40 µL of 0.1% (v/v) formic acid. The peptide content was estimated by UV light spectral density at 280 nm using an extinction coefficient of 1.1 of 0.1% (g/L) solution that was calculated on the basis of the frequency of tryptophan and tyrosine in vertebrate proteins.

*LC‐MS/MS analysis*. LC‐MS/MS analysis was performed on a timsTOF Promass spectrometry (Bruker) that was coupled to Nanoelute (Bruker). The peptides were loaded onto a C18‐reversed phase analytical column (Thermo Scientific Easy Column, 25 cm long, 75 μm inner diameter, 1.9 μm resin) in 95% buffer A (0.1% Formic acid in water) and separated with a linear gradient of buffer B (99.9% acetonitrile and 0.1% Formic acid) at a flow rate of 300 nL/min. The mass spectrometer was operated in positive ion mode. The electrospray voltage applied was 1.5 kV. Precursors and fragments were analyzed at the TOF detector over a mass range of *m/z* 100–1700. The timsTOF Pro was operated in parallel accumulation serial fragmentation (PASEF) mode, PASEF mode data collection was performed based on the following parameters: Ion mobility coefficient (1/K0) value was set from 0.6 to 1.6 Vs cm^2^; 1 MS and 10 MS/MS PASEF scans. The active exclusion was enabled with a release time of 24 s.

*Raw data analysis*. The MS raw data for each sample were combined and searched using the MaxQuant (*1.6.14*) software for identification and quantitation analysis. Related parameters and instructions are as follows: enzyme, trypsin; max missed cleavages, 2; fixed modifications, Carbamidomethyl (C); variable modifications, oxidation (M); main search, 6 ppm; first search, 20 ppm; MS/MS tolerance, 20 ppm; FDR of protein and peptide ≤ 0.01; peptides used for protein quantification, use razor and unique peptides; protein quantification, LFQ; min. ratio count, 1.

*Differentially expressed protein (DEP) filter*: The protein quantification approach used is label‐free quantification (LFQ), commonly applied for pairwise comparisons across multiple groups. Quantitative proteomics data were analyzed by limma (*3.50.3*) within R (*4.1.3*). The main algorithm is pairwise correction through peptide and protein layers. Protein species with the |log2 fold change | ≥ 0.05 and *p*‐adjust ≤ 0.05 were considered DEP between LED‐red and LED‐white.

#### 2.4.3 Metabolome Analysis

The same frozen leaf tissue powders as those used for transcriptome were used for metabolome analysis. *Sample preparation:* Dissolve 100 mg of frozen leaf powder with 1 mL 40:40:20 of methanol, acetonitrile and water solution, vortex 30 s every 10 min for 3 times in total, followed with 4°C overnight. Following centrifugation at 12,000 g for 10 min, the extracts were filtrated (JTSF0306, 0.22 μm pore size; JINTENG, Tianjin, China) before UPLC‐MS/MS analysis.

The sample extracts were analyzed using a UPLC‐ESI‐MS/MS system. The analytical conditions were as follows: UPLC column, Agilent BEH‐C18 (1.7 μm, 2.1 mm × 100 mm). The mobile phase consisted of solvent A 80:20 methanol and acetonitrile, and solvent B, pure water with 2 mm ammonium acetate. Sample measurements were performed with a gradient program that employed the starting conditions of 95% B balanced column for 3.5 min, a linear gradient to 5% B was programmed at 3.5–30 min, and a composition of 5% B was kept for 5 min. Subsequently, a composition of 95% B was adjusted and kept for 5 min. The flow velocity was set as 0.3 mL per minute. The column oven was set to 35°C; The injection volume was 1 μL.

Mass spectrometry was performed on a Q exactive high‐resolution ESI‐triple quadrupole‐linear ion trap (QTRAP) mass spectrometer (Thermo Fisher Scientific, San Jose, USA) using a heated electrospray ionization (HESI) source for ionization of the target compounds in positive and negative ion modes. The ESI source operation parameters were as follows: ionization voltage, +3.4 kV/−3.5 kV; sheath gas pressure, 35 arbitrary units; auxiliary gas, 10 arbitrary units; source temperature 340°C for positive ion mode and 375°C for negative ion mode. For the compounds of interest, a scan range of *m/z* 100–1000 was chosen.

Totally 1318 metabolites were identified with the QTRAP positive and negative ion modes. Significantly regulated metabolites between groups were determined by *t*‐test (*p* < 0.05) and absolute log2FC (fold change) ≥ 0.5. Of this, 186 different accumulated compounds (DACs) were identified. In these DACs, the upregulated by LED‐red light treatments compared to LED‐white was 129 and the down‐related number was 57. MetaboAnalyst (*5.0*, https://www.metaboanalyst.ca/) was employed for the KEGG enrichment analysis.

### 2.5 Statistical Analysis

Effects of different light treatments on plant growth and physiological traits were evaluated by analysis of variance (ANOVA) with R (*3.4.0*). The difference in parameters among different light treatments was tested by one‐way ANOVA at a 95% confidence level. Multiple comparisons for the difference of chlorophyll and total protein content among the light treatments and the infiltrated and non‐infiltrated plants were tested by two‐way ANOVA following turkey‐HSD at a 95% confidence level.

## 3 Results

### 3.1 The Effect of Different Light Quality on *N. benthamiana* Growth

Five commonly‐accessible light quality conditions, including LED‐white (full spectrum LED), LED‐red (660 nm red + 730 nm far‐red), LED‐blue (450 nm), Flu‐white (full spectrum fluorescence) and Flu‐UVB (Flu‐white + 306 nm UV) were set up for *N. benthamiana* cultivation (Figure 1A,B; Supporting Information S1: Table S1). Seedlings transferred to different light environments for 7 days showed diverse growth patterns (Figure 1C), which were prepared for agroinfiltration. Plants under LED‐white showed the largest leaf area and highest shoot dry weight while seedlings under LED‐blue showed similar leaf area but lower shoot dry weight accumulation compared to LED‐white; Flu‐UVB along with LED‐red showed smaller leaf area and shoot dry weight (Figure 1D,E). Furthermore, the Flu‐white group showed the thinnest leaf (Figure 1F). After 7 days preculture under diverse light qualities, the plant is infiltrated by *Agrobacterium* and a similar impact was observed 4 days post‐infiltration (dpi) (Supporting Information S1: Figure S1A–C).

We compared chlorophyll and total soluble protein content of the infiltrated and non‐infiltrated leaves under five light treatments (Table 1). For the non‐infiltrated plants, leaves grown under LED‐red had the lowest chlorophyll content, while leaves grown under LED‐white and LED‐blue showed the highest content. Compared with non‐infiltrated leaves, agroinfiltration reduced the chlorophyll accumulation of leaves under all light qualities, but the LED‐red group still showed the lowest chlorophyll content. For the non‐infiltrated plants, the lowest total protein content was observed in LED‐red; Flu‐white was a little higher than that of LED‐red but still lower than the other three light treatments. However, the infiltrated plants of five light treatments showed no difference in total protein content (Table 1).

**Table 1:** The effects of different light qualities on chlorophyll and total protein content in both infiltrated and non‐infiltrated *Nicotiana benthamiana* leaves.

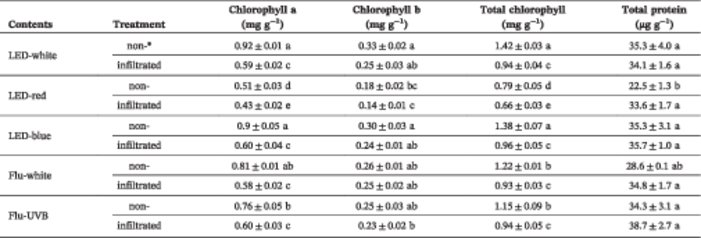

*Note:* Different letters show statistically significant differences within each row (*p* < 0.05). Means ± s.e. (*n* = 4). Non‐*, non‐infiltrated.

### 3.2 Distinct Expression Levels of an Exogenous Protein in Infiltrated Leaves Under Different Light Quality

**Figure 2:**
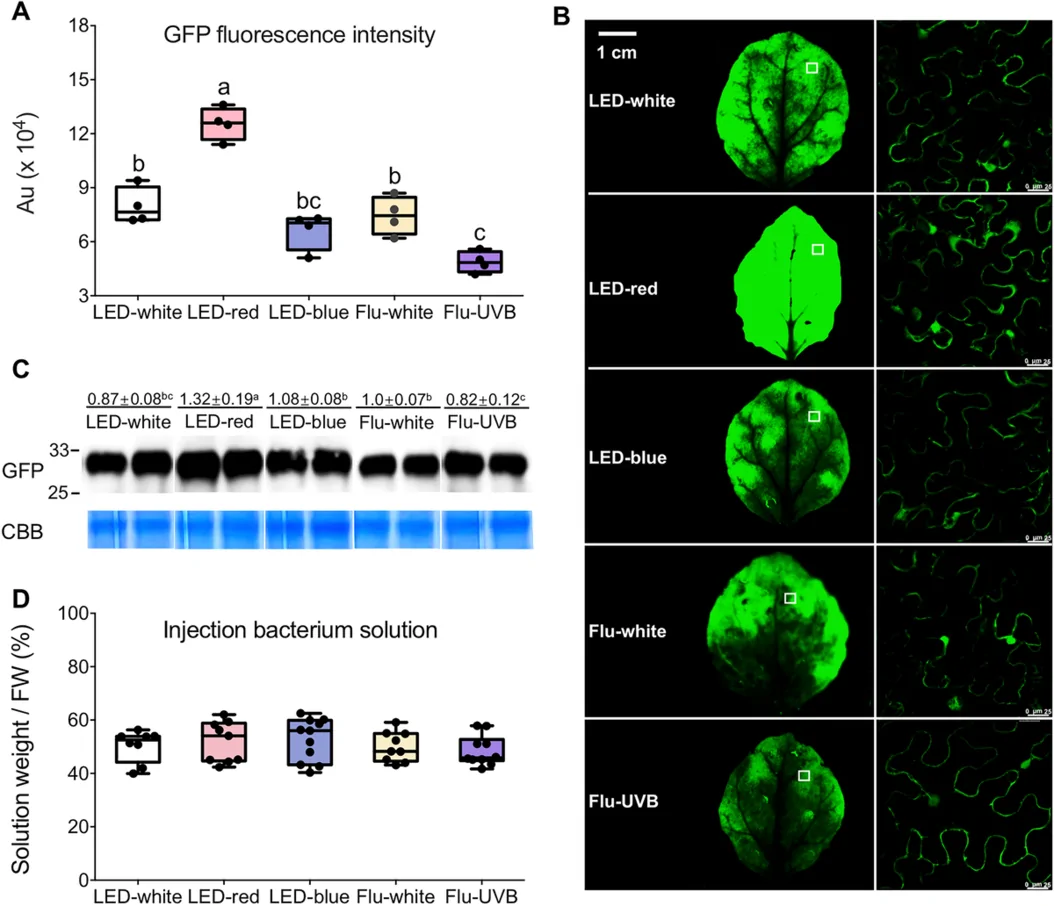
The effect of different light qualities on *Agrobacterium*‐mediated exogenous GFP expression in *Nicotiana benthamiana* leaves. (A) GFP fluorescence intensity measured by protein extract solution. Different letters show statistically significant differences among the light treatments at the same time points (*p* < 0.05) (*n* = 4). (B) Green fluorescence imaging of leaves grown under different light treatments, taken by gel imager (left) and laser scanning confocal microscope (right, Smart gain [HyD]: 10.0%; Zoom: 5.51). (C) Western blot analysis of leaves grown under different light treatments using anti‐GFP antibody. Coomassie blue staining of the blot is shown as a loading control. The numbers represent the relative amount of GFP protein in plants subjected to different light treatments, with all quantitative data normalized to the Flu‐white treatment as the baseline for comparison. Data are presented as the mean ± standard deviation (SD) from four replicates, and the plot includes panels for rep1 and rep2 (Rep3 and rep4 were provided in Supporting Information S1: Figure S4). Different letters show statistically significant differences among the light treatments (*p* < 0.05). (D) The injection quantity of *Agrobacterium* solution per leaf weight in different light treatments plants (*n* = 8–11). [Color figure can be viewed at wileyonlinelibrary.com]

To explore the effect of light quality on *Agrobacterium*‐mediated exogenous protein expression in leaves, pEAQ‐HT‐GFP plasmid was transfected via agroinfiltration and the GFP expression was detected (Figure 2). Four days after agroinfiltration, GFP fluorescence in LED‐red leaves was the strongest among plants grown under different light treatments, 57.4% higher than common LED‐white (Figure 2A,B). These results were further confirmed by the western blot assay (Figure 2C). The injection quantity of *Agrobacterium* solution per leaf weight was controlled to minimize the difference in the initial infiltrated bacteria dosage (Figure 2D).

### 3.3 LED‐Red Promotes Agrobacteria Growth and Plasmid Gene Transcription After Agro‐Infiltration

**Figure 3:**
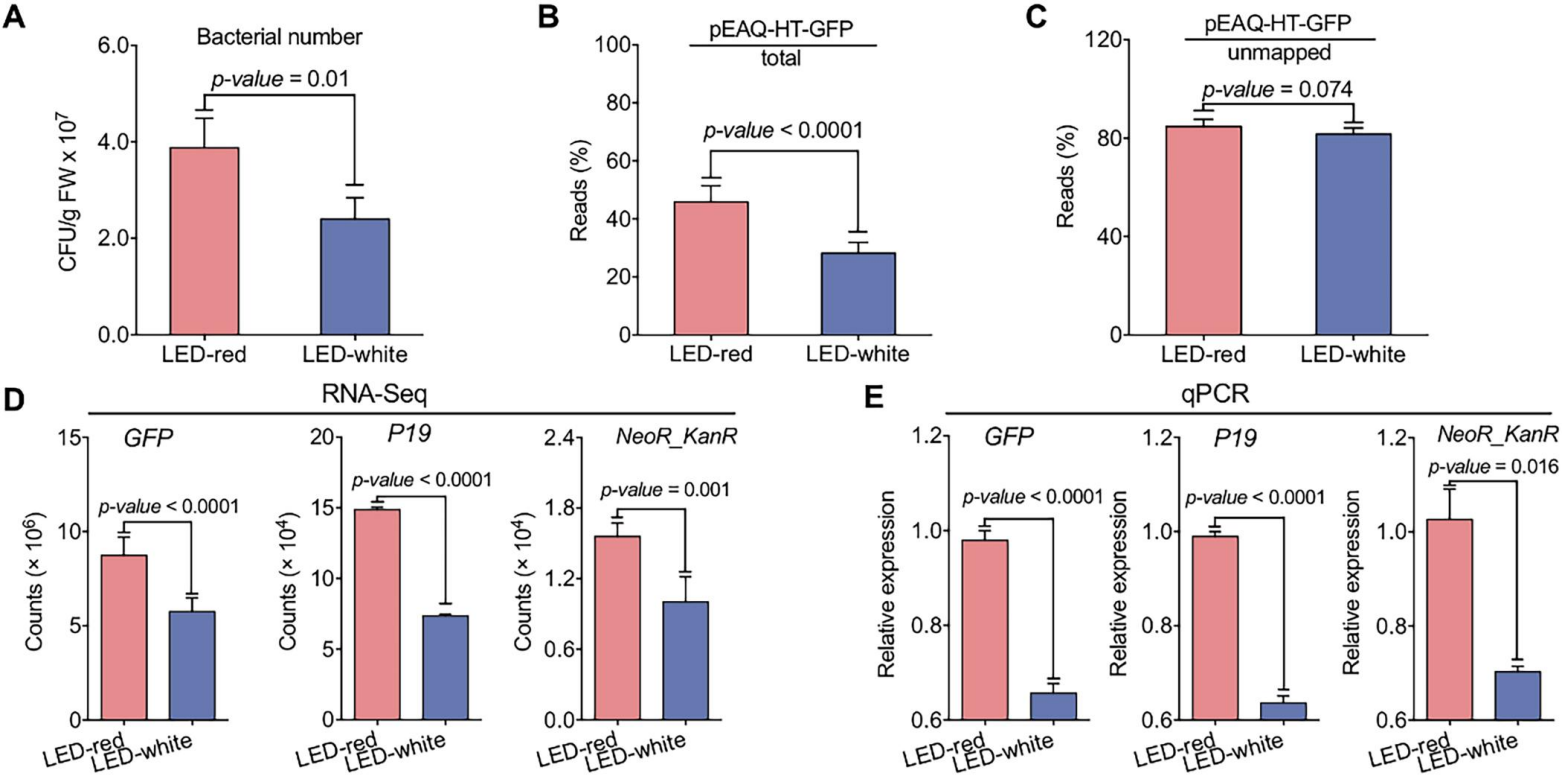
The effect of light qualities on *Agrobacterium* growth and heterogenous gene expression in *Nicotiana benthamiana* leaves. (A) Bacterial abundance in the mesophyll space of plants grown under LED‐red and LED‐white treatments after 4 dpi. Error bars show means ± s.d. (*n* = 4). The ratios of reads mapped to the plasmid per total reads (B) and reads unmapped to plant genome sequence (C). (D, E) The count number of *GFP*, *P19* and *NeoR_KanR* reads and the transcript levels tested by qRT‐PCR. Error bars show means ± s.d (*n* = 3), *p*‐values were calculated by Student's *t*‐tests. [Color figure can be viewed at wileyonlinelibrary.com]

As LED‐red treated leaves showed the highest exogenous GFP expression, we focus the following study on this group, by comparing with LED‐white as the control (Figure 1B). Though the initial injected dosages remained consistent across treatments (Figure 2D), the abundance of agrobacteria at 4 dpi revealed notable differences. Analyses of agroinfiltrated leaf tissue grinding and coating plate assays indicated significantly higher retention of bacteria in the mesophyll space of plants grown under LED‐red light compared to those subjected to other light qualities (Figure 3A; Supporting Information S1: Figure S3).

To compare the expressions of transgene and *N. benthamiana* chassis genes under different light qualities, a comparative transcriptomic analysis was performed between post‐infiltration leaves under LED‐red and LED‐white light treatments. The total filtered clean paired‐reads were classified as ‘mapped to *N. benthamiana* genome’ and ‘unmapped to *N. benthamiana* genome’ (Supporting Information S1: Figure S5), and the ‘unmapped to *N. benthamiana* genome’ part was further mapped to pEAQ‐HT‐GFP plasmid sequence. There were more paired‐reads mapped to the plasmid sequence in LED‐red (41%–55%, *n* = 3) than LED‐white (24%–33%, *n* = 3) (Figure 3B). Vast majority of reads unmapped to *N. benthamiana* genome were mapped the plasmid sequence (ranging from 82% to 88%), but this ratio showed no significant difference between two treatments (Figure 3C).

The exact counts of *GFP* reads from the LED‐red plant were 52% higher than that of LED‐white (Figure 3D), and this expression level was further proved by qRT‐PCR (Figure 3E), but the normalized TPM values between two light treatments showed no difference (Supporting Information S1: Figure S6A). Another two genes from pEAQ plasmid, RNA silencing suppressor *P19* and antibiotic resistance genes *NeoR_KanR* (selection marker) also showed higher counts number under LED‐red than that under LED‐white (Figure 3D). Those results were further confirmed by qRT‐PCR (Figure 3E).

### 3.4 Multi‐Omics Analysis for *N. benthamiana* Plants Grown Under Led‐Red and LED‐White

**Figure 4:**
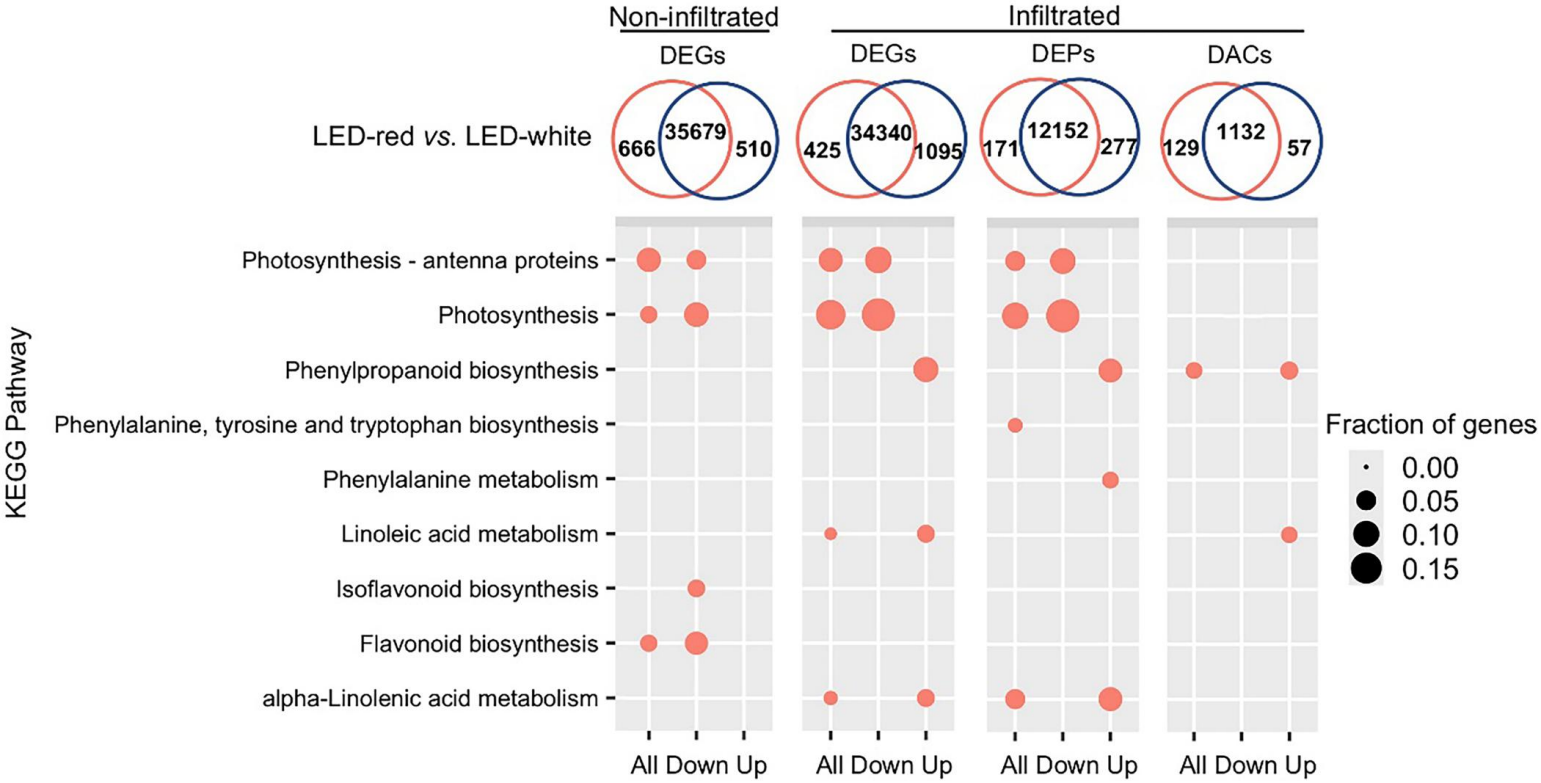
Multi‐omics analysis revealed the difference between *Nicotiana benthamiana* leaves grown under LED‐white and LED‐red. Venn diagrams show the number of upregulated DEGs, DEPs and DACs by *LED‐red (left) or LED‐white (right)*. The bubble plot represents the KEGG pathway analysis of DEGs, DEPs and DACs by the comparisons between LED‐red versus LED‐white. KEGG pathways with a *p*‐value < 0.05 for at least one treatment contrast are included in the figure. The bubble size indicates the proportion of genes, proteins or compounds relative to the total background numbers. [Color figure can be viewed at wileyonlinelibrary.com]

To further explore the potential mechanism underlying the higher exogenous gene expression in plants grown under LED‐red, comparative transcriptomics, proteomics and nontarget metabolomics analyses were performed between agro‐infiltrated leaves from LED‐red and LED‐white environments. A total of 35,860 genes and 12,600 proteins were identified in the assays, whereas the majority of the differentially expressed genes (DEGs) and proteins (DEPs) under LED‐red were downregulated (72% of DEGs; 62% for DEPs) in the comparison of LED‐white (Figure 4). KEGG enrichment analysis of DEGs and DEPs revealed that the relatively upregulated terms were enriched on phenylpropanoid biosynthesis and alpha‐Linolenic acid metabolism, while the downregulated terms were enriched on photosynthesis‐antenna proteins and photosynthesis pathways (Figure 4; complete KEGG results were shown in Supporting Information S1: Figure S7).

However, the transcriptomic comparison for the non‐infiltrated *N. benthamiana* showed that the relatively downregulation including flavonoids biosynthesis in the LED‐red group (Figure 4; complete KEGG results were shown in Supporting Information S1: Figure S8B), which means the agroinfiltration changes the phenylpropanoid biosynthesis pattern, especially flavonoids biosynthesis in *N. benthamiana* chassis.

Metabolome analysis was also performed for the same samples of proteome and transcriptome. We identified 186 DACs in the comparison of LED‐red with LED‐white (Figure 4). Correspondingly, the enrichment analysis of DACs results also showed that infiltrated leaves grown under LED‐red accumulated more compounds belonging to phenylpropanoid biosynthesis and linolenic acid metabolism terms (Figure 4; complete KEGG results were shown in Supporting Information S1: Figure S8A).

### 3.5 Regulation of Phenylpropanoid Pathway Under Different Light Quality

**Figure 5:**
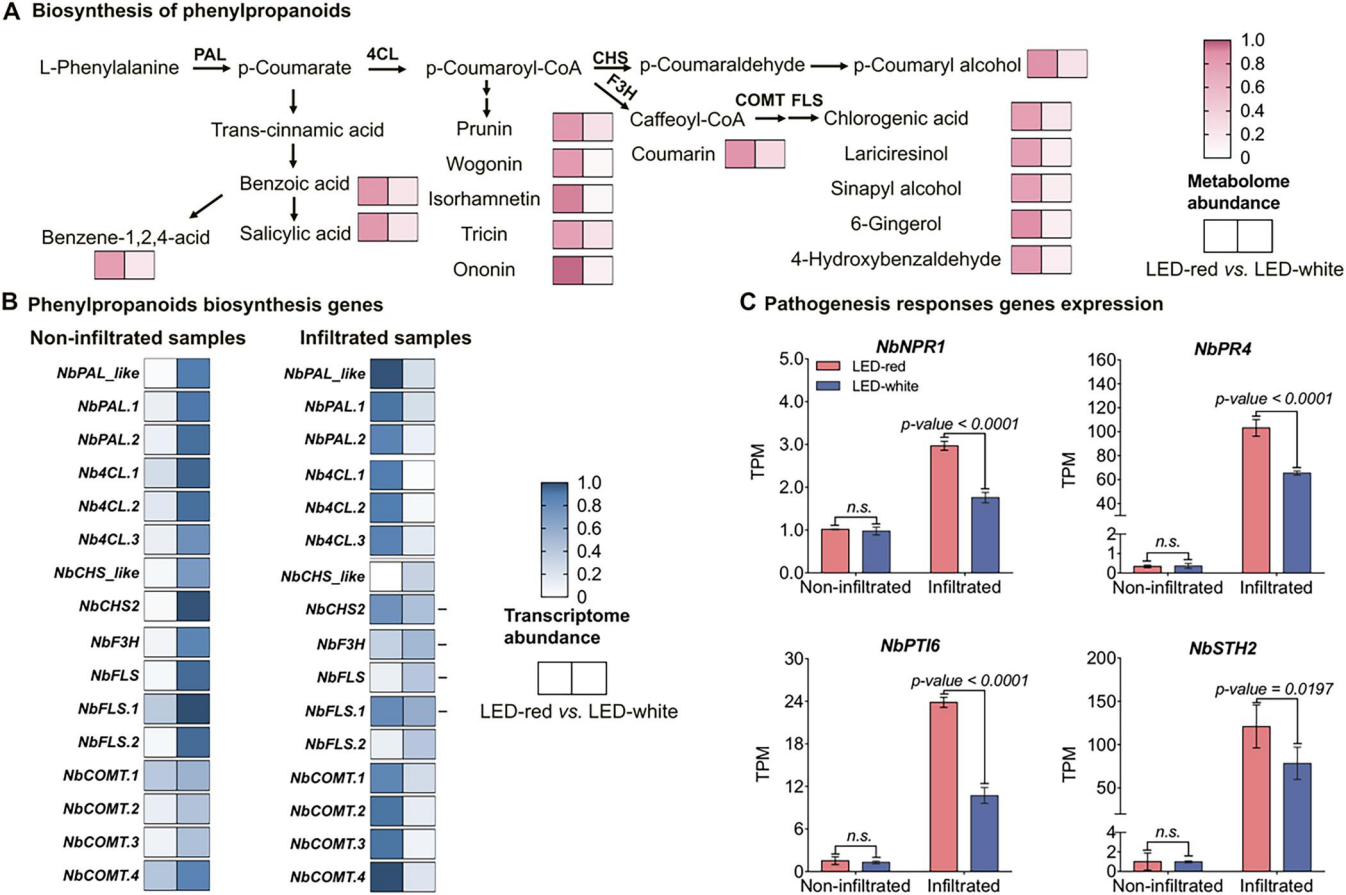
Comparisons of the phenylpropanoid biosynthesis responses at the metabolic or transcriptomic level of the infiltrated and non‐infiltrated leaves grown under LED‐red and LED‐white. (A) Comparison of metabolic abundances of phenylpropanoids of infiltrated *samples*. (B) Comparison of the relative transcriptomic abundances of phenylpropanoids biosynthesis genes of infiltrated and non‐infiltrated *samples*. The short horizontal lines behind the color blocks indicate that the expression levels of the gene are not significantly different. (C) The expressions of key regulator gene of SA *NbNPR1* and pathogenesis‐related genes *NbPTI6*, *NbSTH2* and *NbPR4* in infiltrated and non‐infiltrated leaves. Every metabolite and transcript feature abundance of two treatments in the heatmap were normalized to range from 0 to 1. Error bars show means ± s.d. (*n* = 3), *p*‐values were calculated by Student's *t*‐tests. [Color figure can be viewed at wileyonlinelibrary.com]

Previous studies have reported that the absence of blue light results in reduced accumulation of phenylpropanoids in plants (Siipola et al. 2015; Taulavuori et al. 2016, 2018). But in our comparison, we found 15 phenols and flavanols that were more accumulated in LED‐red, including SA and benzoic acid (Figure 5A), which may be caused by a higher number of *Agrobacterium* after infiltration compared to LED‐white (Figure 3A). This outcome is justifiable given that the accumulation of SA is affected by an increase in the the upstream phenylpropanoid pathway (Sang et al. 2020; Yadav et al. 2020).

The main phenylpropanoids biosynthesis genes including phenylalanine ammonia lyase (*NbPAL*), 4‐coumarate: CoA ligase (*Nb4CL*), chalcone synthase (*NbCHS*), flavanone 3‐hydroxylase (*NbF3H*), catechol‐*O*‐methyltransferase (*NbCOMT*) and flavonol synthase (*NbFLS*) from flavonoids biosynthesis pathway showed higher expression in LED‐red plants than LED‐white, while their expression pattern was opposite in the non‐infiltration leaves grown under those two light quality treatments (Figure 5B). In addition, the SA signaling gene including non‐expressor of pathogenesis‐related gene (*NbNPR1*), pathogenesis‐related genes transcriptional activator PTI6‐like gene (*NbPTI6*), pathogenesis‐related protein STH2‐like gene (*NbSTH2*) and pathogenesis‐related protein PR‐4A gene (*NbPR4*) showed the higher expression response to the agroinfiltration in LED‐red plant, but no significant difference was observed in the non‐infiltrated samples (Figure 5C).

Jasmonic acid precursors such as α‐linolenic acid and 12‐oxo‐phytodienoic acid (12‐OPDA) were also higher accumulated under LED‐red (Supporting Information S1: Figure S9). Both transcriptome and proteome analyses indicated that, in comparison to LED‐white, the expression of genes involved in the biosynthesis pathway of jasmonic acid within chloroplasts was lower under LED‐red treatment. Conversely, genes responsible for functions within peroxisomes exhibited higher expression levels (Supporting Information S1: Figure S9B,C). The variance in expression associated with subcellular location could potentially be attributed to the diminished chlorophyll content observed in LED‐red treatment (Table 1).

### 3.6 Higher Taxadiene Production in *N. benthamiana* Leaves Grown Under LED‐Red

**Figure 6:**
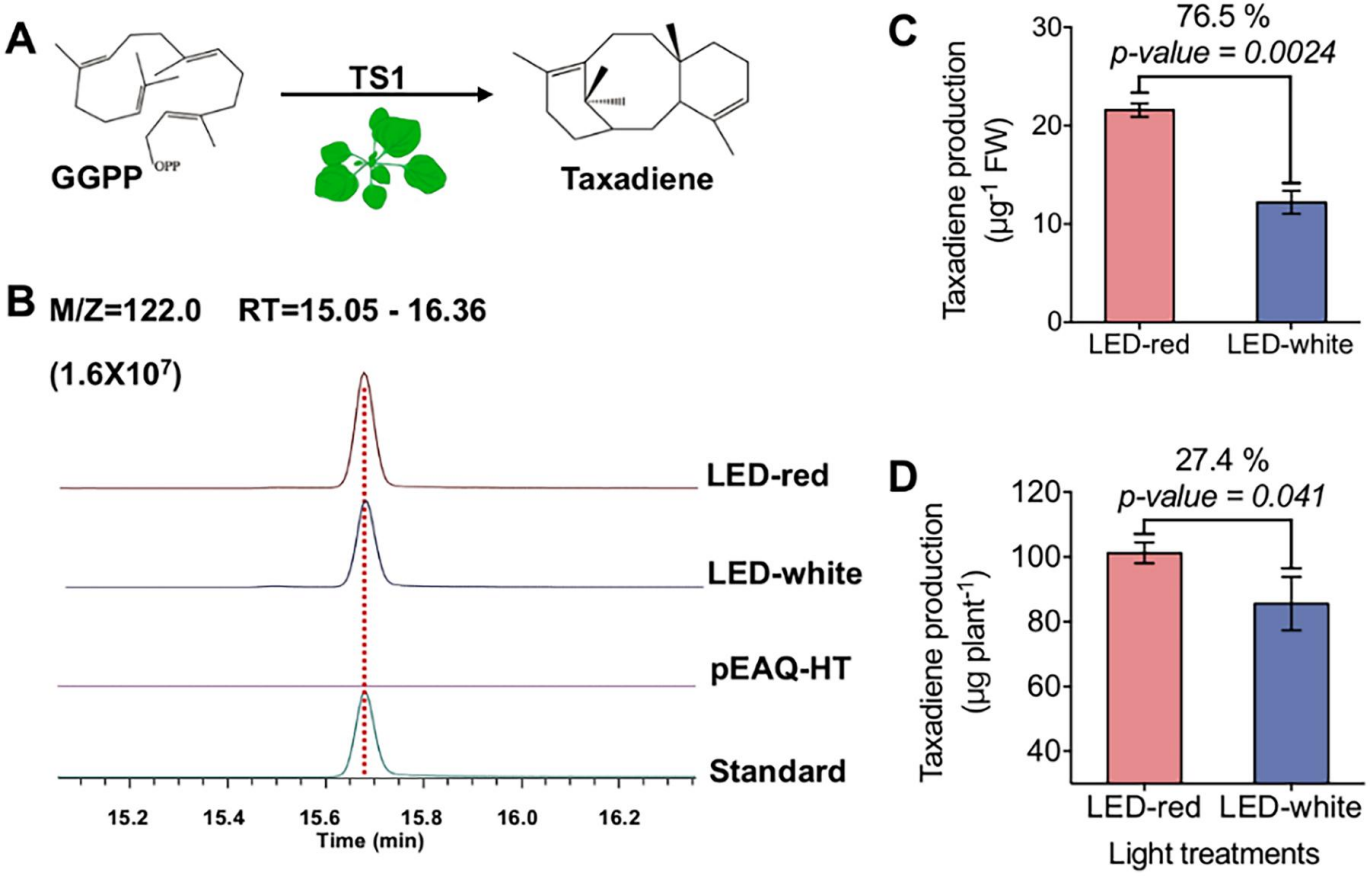
(A) *Agrobacterium*‐mediated taxadiene biosynthesis catalyzed by *TS1* in *Nicotiana benthamiana* leaves grown under two light treatments at the 6 dpi. (B) The taxadiene content was determined by GC‐MS with standard. The taxadiene production of (C) per leaf fresh weight and (D) per plant weight. Numbers represent the percentages of taxadiene production increased by LED‐red relative to that of LED‐white. Error bars show ± s.e. (*n* = 3), *p*‐values were calculated by Student's *t*‐tests. [Color figure can be viewed at wileyonlinelibrary.com]

To further confirm the promotion effect of LED‐red light quality on *Agrobacterium*‐mediated transient transgene expression, we infiltrated leaves from *N. benthamiana* grown under different light conditions with the agrobacteria harboring taxadiene synthetase gene *TS1* to produce taxadiene, the key precursor of chemical therapy medicine paclitaxel (Figure 6A). The result showed that plant grown under LED‐red produced the 76.5% higher taxadiene content than that of LED‐white per fresh weight (Figure 6B,C; Supporting Information S1: Figure S10). Although lower shoot dry weight was observed, the total taxadiene product per plant from the LED‐red group was still 27.4% higher than that of LED‐white treatment (Figure 6D).

## 4 Discussion

Previous studies have reported that environmental factors like light intensity (Fujiuchi et al. 2021; Zhang et al. 2023) or temperature (Fullner and Nester 1996; Dillen et al. 1997; Jung et al. 2015; Matsuda et al. 2019) regulate the heterologous synthesis efficiency. We hypothesized that the light quality may also have the ability to enhance the expression rate due to its close relation to plant growth and immunity. Our results demonstrate that plant responses to different light qualities significantly influence the efficiency of heterologous synthesis for both protein (GFP, Figure 2; Supporting Information S1: Figure S2) and metabolite products (taxadiene, Figure 6; Supporting Information S1: Figure S10), and the combination of red and far‐red light being the most effective option.

*N. benthamiana* exhibited a characteristic phenotype in response to red and far‐red light, characterized by elongated petioles and smaller leaves compared with LED‐white light treatment (Figure 1C), which is consistent with previously reported plant morphological response to this light quality (Demotes‐Mainard et al. 2016). These effects on plant growth remained consistent before and after agroinfiltration (Figure 1D–F; Supporting Information S1: Figure S1). Previous studies have demonstrated that leaves under monochromatic red light or absence of blue light results in diminished photosynthetic efficiency in cucumber (Hogewoning et al. 2010; Trouwborst et al. 2016), tomato (Zhang et al. 2020) and rice (Matsuda et al. 2004). The observed reduction of chlorophyll content under red and far‐red light was also consistent with the decreased photosynthetic activity when compared with the LED‐white light treatment (Table 1), as the lower expressed DEGs and DEPs enriched to photosynthesis pathways (Figure 4; Supporting Information S1: Figures S7 and S8). Lower chlorophyll content is associated with weaker photosynthetic capacity and lower rubisco levels (Medford and Sussex 1989). While Rubisco constitutes approximately 50% of leaf total soluble protein (Ellis 1979), reducing its content is one way to enhance the proportion of exogenously expressed proteins in total soluble protein (Robert et al. 2015). Therefore, the weak photosynthetic capacity resulting from red and far‐red light treatments in this study may provide a great opportunity for the higher accumulation of exogenously expressed proteins.

Photosynthesis plays a pivotal role in sustaining energy supplies and, consequently, is integral to the plant's defense against pathogens. Reduced non‐photochemical quenching of photosynthesis correlates with decreased responsiveness to pathogen‐associated molecular pattern‐triggered immunity in *A. thaliana* (Göhre et al. 2012) while silencing five components of photosystem II in *N. benthamiana* not only hampers photosynthesis but also elevates susceptibility to turnip mosaic virus (Manfre et al. 2011). In this study, the diminished photosynthetic activity induced by red and far‐red light in leaves may provide a physiological basis for the increased survival rate of *Agrobacterium*, possibly due to the weaker plant resistance to *Agrobacterium* infection before invasion.

Plant immune system inhibits pathogen growth and contributes to shaping a healthy microbial community in the plant body. Plants must appropriately respond to both microbial signals and environmental factors, and thus, proper integration of these inputs at local and systemic levels is crucially important. The plant immune system suppresses pathogen growth and helps establish a healthy microbial community within the plant. To achieve this, plants must accurately respond to microbial signals and environmental factors, making the effective integration of these inputs at both local and systemic levels essential (Nobori and Tsuda 2019). After *Agrobacterium* are infiltrated into leaves, host plants attempt to inhibit bacterial growth and burst expression of transgene. Flavonoids are crucial secondary metabolites that play a vital role in plant defense against pathogens (Daayf et al. 2012). Blue light deficiency light condition generally results in less phenylpropanoids, especially flavonoids accumulation in plant blades (Taulavuori et al. 2016, 2018; Zhang et al. 2020), but flavonoids are known to stimulate the expression of *A. tumefaciens* virulence genes (Stachel et al. 1985; Brencic et al. 2004). We observed that for the non‐infiltrated *N. benthamiana leaves, DEGs* from the leaves grown under red and far‐red light were downregulated in flavonoids biosynthesis compared to LED‐white (Figure 4), including the main biosynthesis genes *NbPAL*, *Nb4CL*, *NbCHS*, *NbF3H*, *NbCOMT* and *NbFLS* (Figure 5B). However, infiltrated leaves showed higher expression of biosynthetic genes and flavonoids accumulation in red and far‐red light treatment compared to LED‐white (Figure 5B). We inferred that the red and far‐red light treatment reducing flavonoid synthesis of leaves of before infiltration is the basis for the early outbreak of *Agrobacterium*, while the elevated *Agrobacterium* triggered plant defense strategy response after infiltration leading to higher flavonoids compound content.

The accumulation of SA is vital in acquired resistance against pathogens (Shah 2003). As downstream products of the same phenylpropanoid pathway, SA also showed higher content in red and far‐red light treatment (Figure 5A). We also observed that different α‐linolenic acid metabolism between red and far‐red light and LED‐white light treatments from transcriptome to metabolome level, and finally there was higher α‐linolenic acid content in red and far‐red light grown leaves (Supporting Information S1: Figure S9). However, we did not observe a significantly higher accumulation of JA in the red and far‐red light treatment (Supporting Information S1: Figure S9D). JA is recognized as the primary mediator of immune responses against necrotrophic pathogens. Generally, SA and JA are considered antagonistic, with each balancing the effects of the other (Bürger and Chory 2019). However, a previous study also showed that *Ralstonia solanacearum* type‐III effector RipE1 induces both SA and JA accumulation in *N. benthamiana* (Sang et al. 2020), suggesting that two hormones may also act cooperatively in response to bacterium effectors under varying conditions.

Taken together, our findings suggest that the increased presence of *Agrobacterium* under the red and far‐red light quality (Figure 3A; Supporting Information S1: Figure S3) was attributed to the compromised immune function resulting from the aforementioned photosynthetic dysfunction and weak flavonoid accumulation. Consequently, the elevated bacterium counts corresponded to a relatively greater transcription of plasmid‐converted templates (Figure 3B,C), increased protein accumulation (Figure 2; Supporting Information S1: Figure S2), and ultimately higher catalytic product accumulation (Figure 6; Supporting Information S1: Figure S10). The ratios of reads that mapped to the plasmid sequence to total reads ranged from 24% to 55% (Figure 3B: higher in LED‐red, lower in LED‐white) in this study, which means there were plasmid‐driven expression eruptions at the transcriptome level. The uniform TPM values observed for plasmid‐converted GFP expression under both LED‐red and LED‐white light treatments (Supporting Information S1: Figure S6A) further substantiate that disparities in gene expression are primarily attributed to differences in template quantities.

Heterologous biosynthesis of taxadiene was reconstructed and further modified in both chloroplast (Li et al. 2019) and cytosolic (De‐La‐Peña and Sattely 2021) in *N. benthamiana*. Aside from molecular and genetic operation of vectors and genes, this new cultivate method provides the efficiency enhancement of heterogenous taxadiene production (increased 76.5%) when using LED‐red growth condition (Figure 6). Those above methods may be combined for further loop optimization of protein and metabolite production in plant chassis.

**Figure 7:**
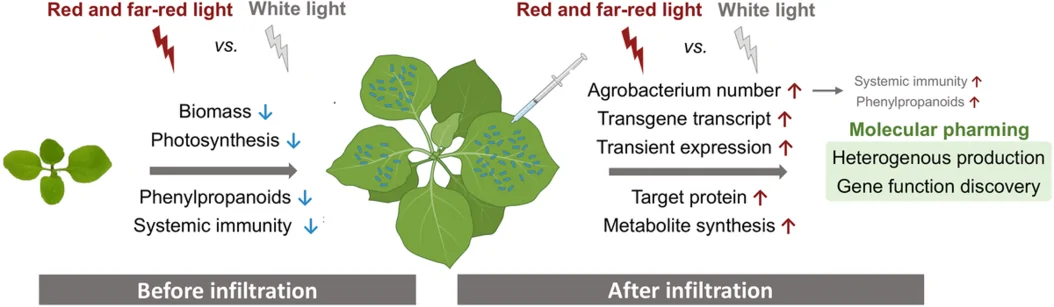
A working model of red light and far‐red light combination treats *Nicotiana benthamiana* chassis is more suitable for *Agrobacterium‐mediated* transient transformation and heterologous synthesis. [Color figure can be viewed at wileyonlinelibrary.com]

In this study, we offered an optimal growth environment to enhance the expression of transgenes introduced through the *Agrobacterium*‐mediated transformation of *N. benthamiana*. Red and far‐red light stands out as a ‘safe’ option for *Agrobacterium* inoculation within *N. benthamiana* leaves, allowing for higher retention of bacteria and the efficient expression of exogenous genes (Figure 7). Consequently, the light quality can be considered a valuable external factor for optimizing the product rate and gene function discovery in transient expression systems. The plant chassis for efficient heterologous product synthesis obtained through light environment modification can further accelerate the molecular pharming industry.

## Conflicts of Interest

The authors declare no conflicts of interest.

## Supporting information

Supplementary files resubmit clean.

## Data Availability

The data that support the findings of this study are available on request from the corresponding author. The data are not publicly available due to privacy or ethical restrictions.
