# Supplementary material for: Multi‐Omics Analyses Reveal the Red and Far‐Red Light Combination Enhancing Heterologous Protein and Metabolite Production in Nicotiana benthamiana
Source: Plant Cell Environ. 2025 Apr 23;49(10):7435–48. doi: 10.1111/pce.15573 (PMC13551632; doi:10.1111/pce.15573)
Supplement: Supplementary file 1 [file PCE-49-7435-s001.docx]

**Multi-omics analyses reveal the red and far-red light combination enhancing heterologous protein and metabolite production in *Nicotiana benthamiana***

## Supplementary Figures

**Supplementary figure 1** *N. benthamiana* plants’ growth in responses to different light treatments. leaf area (A), shoot dry weight (B) and specific leaf area (C) of the plants grown under different light treatments at 4 dpi (days post-infiltration). Different letters show statistically significant differences among the light treatments in the same time points (*P* < 0.05). Error bars show ± s.e. The experiment was repeated twice with similar results.

**Supplementary figure 2** Green fluorescence imaging of leaves grown under different light treatments at the 4 dpi.

**Supplementary figure 3** Bacterial abundance in five light quality treatments plants at the 4 dpi.

**Supplementary figure 4** Western blot of leaves grown under different light treatments with anti-GFP. Coomassie blue staining of the blot is shown as loading control, this plot showed the rep3 and rep4.

**Supplementary figure 5** The ratios of filtered clean paired-reads that unmapped to *N. benthamiana* genome to total filtered clean paired-reads.

**Supplementary figure 6** The relative expression of transgenes represented by TPM on the plasmid insertion sequence.

**Supplementary figure 7** Bubble plot represents the KEGG pathway analysis of DEGs and DEPs by the comparisons between LED-red *vs.* LED-white. KEGG pathways with *P-value* < 0.05 for at least one treatments contrast are included in the figure. Color represents *P-value*s, and the bubble size represents the ratio of genes, proteins or compounds in term to total background numbers.

**Supplementary figure 8** Comparisons of the phenylpropanoid compounds biosynthesis responses at transcriptomic or metabolic level of the infiltrated and non-infiltrated leaves grown under LED-red and LED-white. (A) KEGG enrichment analysis of the DACs of infiltrated samples. (B) KEGG enrichment analysis of the DEGs of non-infiltrated samples. Color represents *P-value*s, and the bubble size represents the ratio of genes, proteins or compounds in term to total background numbers.

**Supplementary figure 9** (A) The synthetic pathway of jasmonic acid in plants and the specific subcellular locations of different synthetic steps. Comparison of the relative transcriptomic (B), proteomic (C) and metabolic (D) abundances of key genes for jasmonic acid biosynthesis of infiltrated samples under LED-red and LED-white.

**Supplementary figure 10** (A) *Agrobacterium*-mediated taxadiene biosynthesis catalyzed by TS1 in *N. benthamiana* leaves grown under five light treatments at the 6 dpi. (B) The taxadiene content was determined by GC-MS with standard. The taxadiene production of (C) per leaf fresh weight and (D) per plant weight. Different letters show statistically significant differences among the light treatments in the same time points (*P* < 0.05). Error bars show ± s.e. (n = 4).

**Supplementary figure 11** Structure of pEAQ-HT-eGFP plasmid in this study. The T-DNA segment ranges from RB T-DNA repeat to LB T-DNA repeat, which includes the interest gene (*eGFP*), *P19* and *NeoR/KanR*.

| Supplementary Table 1 The light spectrum distribution of five light treatments | | | | | | |
| --- | --- | --- | --- | --- | --- | --- |
| Light treatments | UVB^*^ (%) | Blue^*^ (%) | Green^*^ (%) | Red^*^ (%) | Far-red^*^ (%) | Total PFD^*^  (µmol m^-2^ s^-1^) |
| Flu-white | 0 | 30.2 | 42.2 | 25.3 | 2.3 | 171.6±13.6 |
| Flu-UVB | 0.8 | 30.9 | 41.9 | 25.2 | 2.2 | 178.1±8.3 |
| LED-Blue | 0 | 49.2 | 31.4 | 17.8 | 1.7 | 185.5±9.7 |
| LED-White | 0 | 19.6 | 38.8 | 40.6 | 1.1 | 185.2±14.7 |
| LED-Red | 0 | 0 | 0 | 71.4 | 28.6 | 183.4±11.2 |
| ^*^UVB light, 280-315 nm; ^*^Blue light, 400-500 nm; ^*^Green light, 500-600 nm; ^*^Red light, 600-700 nm; ^*^Far-red light, 700-750 nm; ^*^PFD, Photon flux density, PFD value means±SE, n = 9. Supplementary Table 2 List of primers used for qRT-PCR in this study. \| \|; Gene symbol \| Forward primer (5'-3') \| Reverse primer (5'-3'); *tubA1* \| CAAGACTAAGCGTACCATCCA \| TTGAATCCAGTAGGGCACCAG; *GFP* \| GAGGTGAAGTTCGAGGGCGA \| AGCTTGTGCCCCAGGATGTT; *P19* \| GCTTCACTGCACAGAGTCCT \| GAGACCCTCCAGAAACGGTG; *NeoR_KanR* \| TGCCTGCTTGCCGAATATCA \| ATATCACGGGTAGCCAACGC | | | | | | |

| Supplementary Table 3 Sequences of genes on plasmid. | |
| --- | --- |
| Gene symbol | Sequence |
| *GFP* | ATGGTGAGCAAGGGCGAGGAGCTGTTCACCGGGGTGGTGCCCATCCTGGTCGAGCTGGACGGCGACGTAAACGGCCACAAGTTCAGCGTGTCCGGCGAGGGCGAGGGCGATGCCACCTACGGCAAGCTGACCCTGAAGTTCATCTGCACCACCGGCAAGCTGCCCGTGCCCTGGCCCACCCTCGTGACCACCCTGACCTACGGCGTGCAGTGCTTCAGCCGCTACCCCGACCACATGAAGCAGCACGACTTCTTCAAGTCCGCCATGCCCGAAGGCTACGTCCAGGAGCGCACCATCTTCTTCAAGGACGACGGCAACTACAAGACCCGCGCCGAGGTGAAGTTCGAGGGCGACACCCTGGTGAACCGCATCGAGCTGAAGGGCATCGACTTCAAGGAGGACGGCAACATCCTGGGGCACAAGCTGGAGTACAACTACAACAGCCACAACGTCTATATCATGGCCGACAAGCAGAAGAACGGCATCAAGGTGAACTTCAAGATCCGCCACAACATCGAGGACGGCAGCGTGCAGCTCGCCGACCACTACCAGCAGAACACCCCCATCGGCGACGGCCCCGTGCTGCTGCCCGACAACCACTACCTGAGCACCCAGTCCGCCCTGAGCAAAGACCCCAACGAGAAGCGCGATCACATGGTCCTGCTGGAGTTCGTGACCGCCGCCGGGATCACTCTCGGCATGGACGAGCTGTACAAGTGA |
| *TS1* | ATGGCTCAGCTCTCATTTAATGCAGCGCTGAAGATGAATGCATTGGGGAACAAGGCAATCCACGATCCAACGAATTGCAGAGCCAAATCTGAGGGCCAAATGATGTGGGTTTGCTCCAAATCAGGGCGAACCAGAGTAAAAATGTCGAGAGGAAGTGGTGGTCCTGGTCCTGTCGTAATGATGAGCAGCAGCACTGGCACTAGCAAGGTGGTTTCCGAGACTTCCAGTACCATTGTGGATGATATCCCTCGACTCTCCGCCAATTATCATGGCGATCTGTGGCACCACAATGTTATACAAACTCTGGAGACACCATTTCGTGAGAGTTCTACTTACCAAGAACGGGCAGATGAGCTGGTTGTGAAAATTAAAGATATGTTCAATGCGCTCGGAGACGGAGATATCAGTCCGTCTGCATACGACACTGCGTGGGTGGCGAGGGTGGCGACCATTTCCTCTGATGGATCTGAGAAGCCACGGTTTCCTCAGGCCCTCAACTGGGTTTTAAACAACCAGCTACAAGACGGATCATGGGGTATCGAATCGCACTTTAGTTTATGCGATCGATTGCTTAACACGATCAATTCTGTTATCGCTCTCTCGGTTTGGAAAACAGGGCACAGCCAAGTAGAACAAGGTACTGAGTTTATTGCAGAGAATCTAAGATTACTCAATGAGGAAGATGAGTTGTCCCCGGATTTCGAAATAATCTTTCCTGCTCTGCTGCAAAAGGCAAAATCGTTGGGGATCAATCTTCCTTACGATCTTCCATTTATCAAATATTTGTCGACAACACGGGAAGCCAGGCTTACAGATGTTTCTGCGGCAGCAGACAATATTCCAGCCAACATGTTGAATGCGTTGGAGGGTCTTGAGGAAGTTATTGACTGGAAGAAGATTATGAGGTTTCAAAGTAAAGATGGATCTTTCCTGAGCTCCCCTGCCTCCACTGCCTGTGTACTGATGAATACAGGGGACGAAAAATGTTTCACTTTTCTCAACAATCTGCTGGACAAATTCGGCGGCTGCGTGCCCTGTATGTATTCCATCGATCTGCTGGAACGCCTTTCGCTGGTTGATAACATTGAGCATCTCGGAATCGGTCGCCATTTCAAACAAGAAATCAAAGTAGCTCTTGATTATGTCTACAGACATTGGAGTGAAAGGGGCATCGGTTGGGGCAGAGACAGCCTCGTTCCAGATCTCAACACAACAGCCCTCGGCCTGCGAACTCTTCGCACGCACGGATACGATGTTTCTTCAGATGTTTTGAATAATTTCAAAGATGAAAACGGGCGGTTCTTCTCCTCTGCGGGCCAAACCCATGTCGAATTGAGAAGCGTGGTGAATCTTTTCAGAGCTTCCGACCTTGCATTTCCTGACGAAGGAGCTATGGACGATGCTAGAAAATTTGCAGAACCATATCTTAGAGACGCACTTGCAACAAAAATCTCAACCAATACAAAACTATTCAAAGAGATTGAGTACGTGGTGGAGTACCCTTGGCACATGAGTATCCCACGCCTAGAAGCCAGAAGTTATATTGATTCGTATGATGACGATTATGTATGGGAGAGGAAGACTCTATACAGAATGCCATCTTTGAGTAATTCAAAATGTTTAGAATTGGCAAAATTGGACTTCAATATCGTACAATCTTTGCATCAAGAGGAGTTGAAGCTTCTAACAAGATGGTGGAAGGAATCCGGCATGGCAGATATAAATTTCACTCGACACCGAGTGGCGGAGGTTTATTTTTCATCAGCTACATTTGAACCCGAATATTCTGCCACTAGAATTGCCTTCACAAAAATTGGTTGTTTACAAGTCCTTTTTGATGATATGGCTGACATCTTTGCAACACTAGATGAATTGAAAAGTTTCACTGAGGGAGTAAAGAGATGGGATACATCTTTGCTACATGAGATTCCAGAGTGTATGCAAACTTGCTTTAAAGTTTGGTTCAAATTAATGGAAGAAGTAAATAATGATGTGGTTAAGGTACAAGGACGTGACATGCTCGCTCACATAAGAAAACCCTGGGAGTTGTACTTCAATTGTTATGTACAAGAAAGGGAGTGGCTTGAAGCTGGGTATATACCAACTTTTGAAGAGTACTTAAAGACTTATGCTATATCAGTAGGCCTTGGACCGTGTACCCTACAACCAATACTACTAATGGGTGAGCTTGTGAAAGATGATGTTGTTGAGAAAGTGCACTATCCCTCAAATATGTTTGAGCTTGTATCCTTGAGTTGGCGACTAACAAACGACACCAAAACATATCAGGCTGAAAAGGCTCGAGGACAACAAGCCTCAGGCATAGCATGCTATATGAAGGATAATCCAGGAGCAACTGAGGAAGATGCCATCAAGCACATATGTCGTGTTGTTGACCGGGCCTTGAAAGAAGCAAGCTTTGAATATTTCAAACCATCCAATGATATCCCAATGGGTTGTAAGTCCTTTATTTTTAACCTTAGATTGTGTGTCCAAATCTTTTACAAGTTTATAGATGGGTACGGAATCGCCAATGAGGAGATTAAGGATTATATAAGAAAAGTTTATATTGATCCAATTCAAGTA |
| *P19* | ATGGAACGAGCTATACAAGGAAACGACGCTAGGGAACAAGCTAACAGTGAACGTTGGGATGGAGGATCAGGAGGTACCACTTCTCCCTTCAAACTTCCTGACGAAAGTCCGAGTTGGACTGAGTGGCGGCTACATAACGATGAGACGAATTCGAATCAAGATAATCCCCTTGGTTTCAAGGAAAGCTGGGGTTTCGGGAAAGTTGTATTTAAGAGATATCTCAGATACGACAGGACGGAAGCTTCACTGCACAGAGTCCTTGGATCTTGGACGGGAGATTCGGTTAACTATGCAGCATCTCGATTTTTCGGTTTCGACCAGATCGGATGTACCTATAGTATTCGGTTTCGAGGAGTTAGTATCACCGTTTCTGGAGGGTCTCGAACTCTTCAGCATCTCTGTGAGATGGCAATTCGGTCTAAGCAAGAACTGCTACAGCTTGCCCCAATCGAAGTGGAAAGTAATGTATCAAGAGGATGCCCTGAAGGTACTGAGACCTTCGAAAAAGAAAGCGAGTAA |
| *NeoR_KanR* | ATGATTGAACAAGATGGATTGCACGCAGGTTCTCCGGCCGCTTGGGTGGAGAGGCTATTCGGCTATGACTGGGCACAACAGACAATCGGCTGCTCTGATGCCGCCGTGTTCCGGCTGTCAGCGCAGGGGCGCCCGGTTCTTTTTGTCAAGACCGACCTGTCCGGTGCCCTGAATGAACTGCAGGACGAGGCAGCGCGGCTATCGTGGCTGGCCACGACGGGCGTTCCTTGCGCAGCTGTGCTCGACGTTGTCACTGAAGCGGGAAGGGACTGGCTGCTATTGGGCGAAGTGCCGGGGCAGGATCTCCTGTCATCTCACCTTGCTCCTGCCGAGAAAGTATCCATCATGGCTGATGCAATGCGGCGGCTGCATACGCTTGATCCGGCTACCTGCCCATTCGACCACCAAGCGAAACATCGCATCGAGCGAGCACGTACTCGGATGGAAGCCGGTCTTGTCGATCAGGATGATCTGGACGAAGAGCATCAGGGGCTCGCGCCAGCCGAACTGTTCGCCAGGCTCAAGGCGCGCATGCCCGACGGCGATGATCTCGTCGTGACCCATGGCGATGCCTGCTTGCCGAATATCATGGTGGAAAATGGCCGCTTTTCTGGATTCATCGACTGTGGCCGGCTGGGTGTGGCGGACCGCTATCAGGACATAGCGTTGGCTACCCGTGATATTGCTGAAGAGCTTGGCGGCGAATGGGCTGACCGCTTCCTCGTGCTTTACGGTATCGCCGCTCCCGATTCGCAGCGCATCGCCTTCTATCGCCTTCTTGACGAGTTCTTCTGA |
